# Juvenile Fibroadenoma in a Prepubertal Girl With Idiopathic Precocious Puberty: A Case Report Challenging Hormonal Paradigms

**DOI:** 10.7759/cureus.83779

**Published:** 2025-05-09

**Authors:** Hamza Bensaghir, Asma Abbassi, Chaimae Ben Driss, Mohamed Rami, Mohamed El Amine Bouhafs

**Affiliations:** 1 Department of Pediatric Surgery, Children’s Hospital, Faculty of Medicine and Pharmacy, Mohammed V University, Rabat, MAR

**Keywords:** benign breast lesion, juvenile fibroadenoma, pediatric breast mass, precocious puberty, prepubertal breast mass

## Abstract

Juvenile fibroadenoma (JF) is a rare benign breast tumor that predominantly occurs in adolescent and young adult females. However, its occurrence in prepubertal girls is exceedingly rare and poses diagnostic and therapeutic challenges. We present a case of a six-year-old girl with a rapidly growing breast mass, ultimately diagnosed as juvenile fibroadenoma. This case highlights the importance of considering JF in the differential diagnosis of breast masses in pediatric patients, even in very young children. The clinical presentation, diagnostic workup, and management of JF are discussed, along with a review of the literature to provide insights into this rare condition.

## Introduction

Juvenile fibroadenoma (JF), a distinct variant of benign fibroadenoma, primarily affects younger patients and exhibits rapid growth. Unlike conventional fibroadenomas that predominantly occur in women aged 15-35 years, JF appears more frequently in adolescents and is exceptionally rare in prepubertal children, likely due to immature breast tissue and limited hormonal stimulation. Reported incidence in children under 10 is extremely low [1]. Diagnosing JF in a six-year-old patient represents an exceptional clinical scenario that necessitates thorough evaluation to distinguish it from other pediatric breast masses, including phyllodes tumors, lipomas, and malignant lesions [2].

Effective management of pediatric JF demands a multidisciplinary strategy incorporating pediatricians, radiologists, and surgeons to achieve both accurate diagnosis and optimal treatment. This report expands the limited literature on prepubertal JF cases while offering practical guidance for clinicians.

## Case presentation

Patient presentation

A six-year-old premenarchal girl presented with a rapidly enlarging left breast mass first noted by her parents four weeks earlier. The initially small lesion had progressed significantly, causing visible asymmetry. The patient reported no associated pain, nipple discharge, or breast trauma. She denied systemic symptoms, including fever or weight loss. Both personal and family histories revealed no breast pathology or malignancy.

Physical examination

Examination revealed a 4 cm firm, mobile, non-tender mass in the upper outer quadrant of the left breast. The overlying skin showed no erythema, ulceration, or peau d'orange changes. No contralateral breast masses or lymphadenopathy were detected. The remainder of the examination was normal. Tanner stage was I.

Diagnostic workup

Biological Assessment

The endocrine and tumor marker profiles are given in Table 1. No other abnormalities were noted in the laboratory findings.

**Table 1:** Endocrine and tumor marker profile LH: luteinizing hormone, FSH: follicle-stimulating hormone, β-hCG: beta–human chorionic gonadotropin.

Parameter	Patient Value	Reference Range (Prepubertal)	Interpretation
LH	6.57 IU/L	<0.3–4.9 IU/L	Elevated
FSH	7.89 IU/L	<0.3–4.1 IU/L	Elevated
Estradiol	24.1 pg/mL	<10–20 pg/mL	Mildly elevated
β-hCG	Normal	<2–5 IU/L (age-dependent)	Rules out germ cell tumor
α-Fetoprotein	Normal	<10 ng/mL	Rules out hepatoma

Radiological Assessment

**Figure 1:**
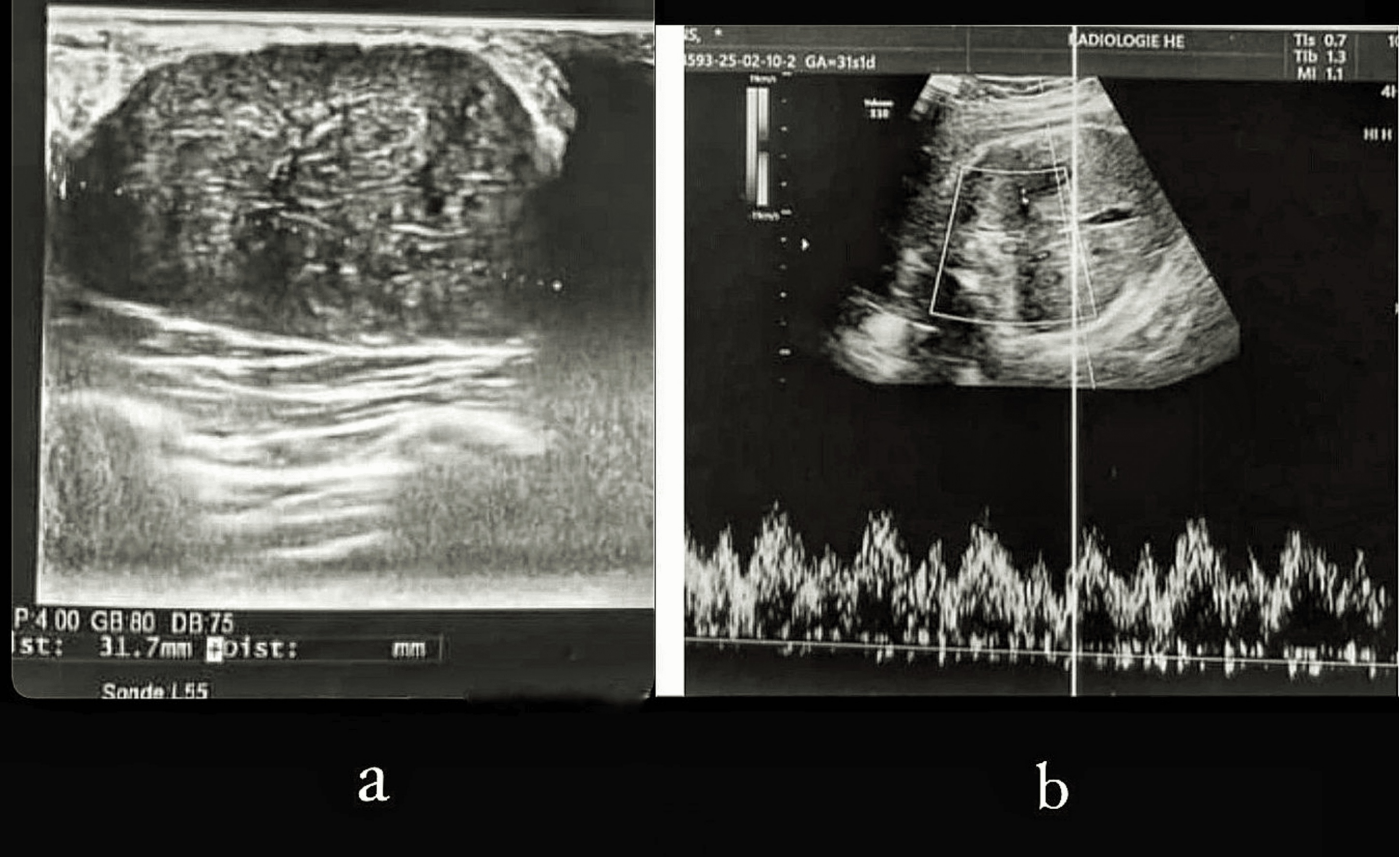
Breast ultrasound images of the present case (a) B-mode ultrasound image showing the mass. (b) Pulsed Doppler ultrasound image showing central vascularization of the mass.

Brain and pituitary MRI was normal. Breast ultrasound (Figure 1) showed hypertrophy of both mammary glands with an oval, well-circumscribed, homogeneous, hypoechoic mass measuring 35 × 18 mm in the upper outer quadrant of the left breast.

Given the patient's young age, mammography was not performed to avoid unnecessary radiation exposure.

Enhanced Endocrine Correlation

The patient's hormonal profile demonstrated elevated LH (6.57 IU/L) and FSH (7.89 IU/L) accompanied by normal estradiol levels (24.1 pg/mL), consistent with idiopathic precocious puberty. Pediatric endocrinologists confirmed the diagnosis following exclusion of CNS pathology through normal pituitary MRI findings. This hormonal pattern, involving elevated gonadotropins without a corresponding estradiol surge, is atypical and highlights possible peripheral or transient activation pathways.

Management approach

**Figure 2:**
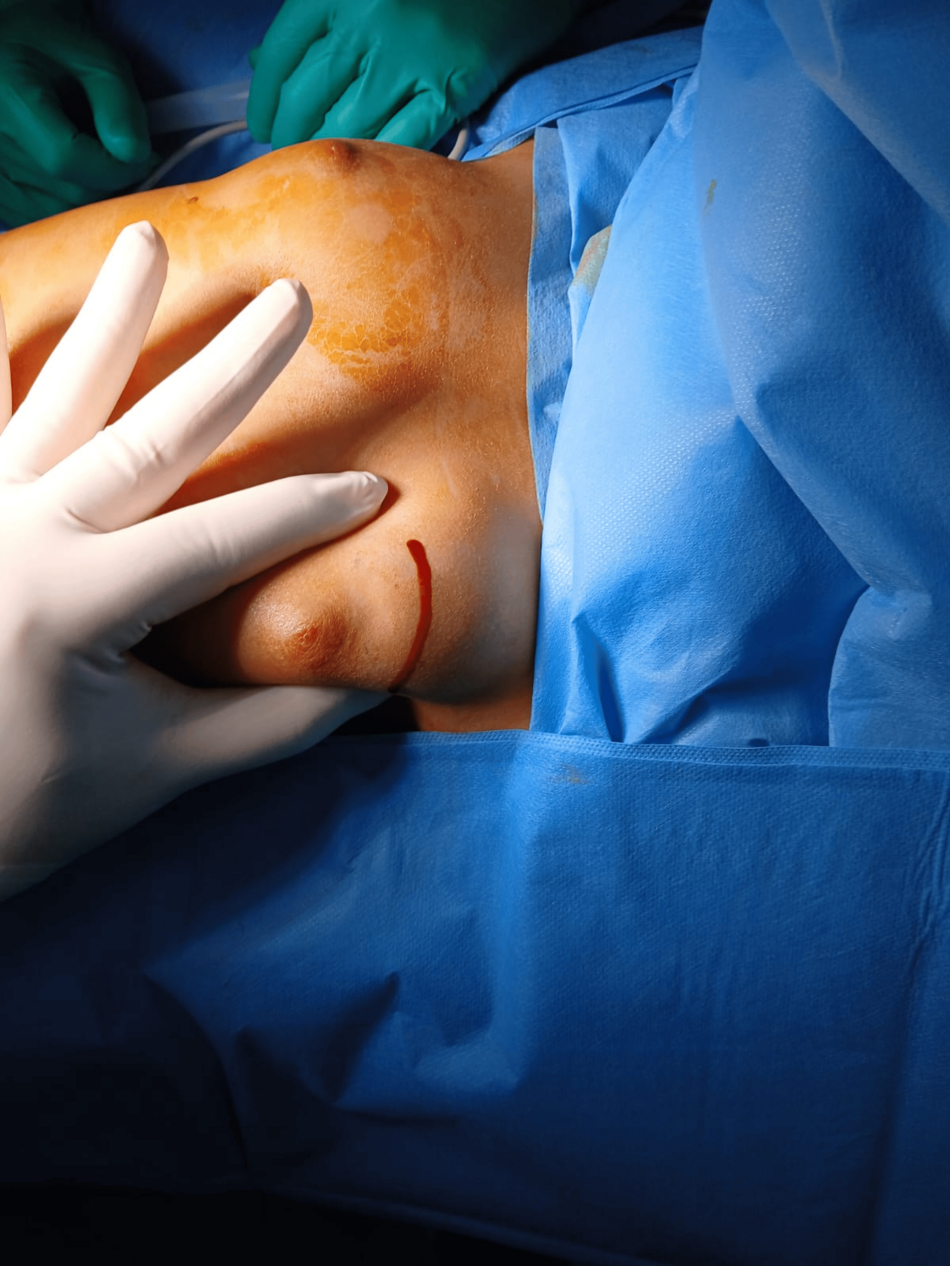
Preoperative image showing the surgical approach used

**Figure 3:**
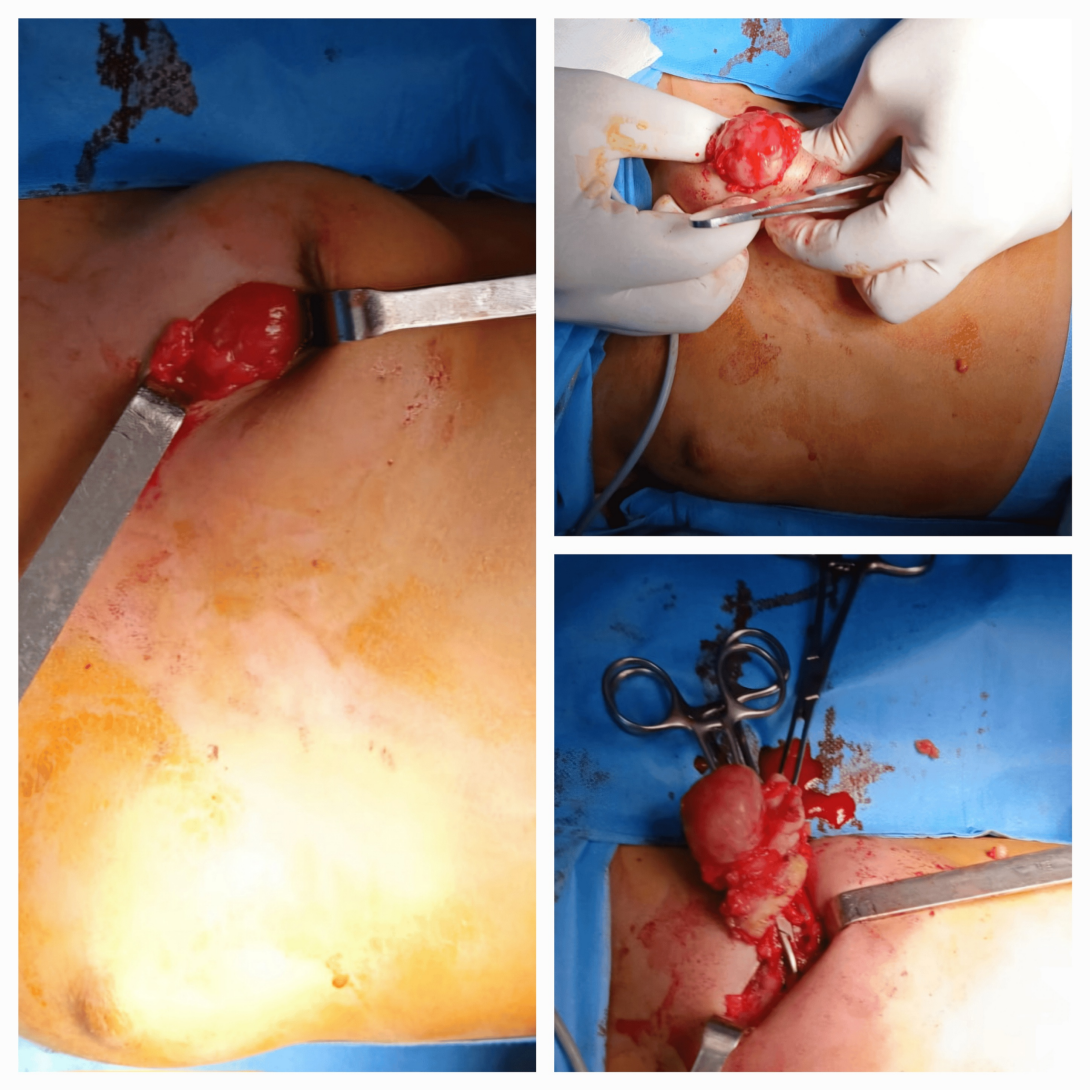
Surgical images showing the stages of resection

Under general anesthesia, a 2 cm transverse incision was created at the upper quadrant junction (Figure 2). A well-demarcated, mobile tumor encased in a thin fibrous capsule was identified (Figure 3). Complete excision was performed with 2 cm margins, selected due to the rapid tumor growth and concern for a potential phyllodes tumor. Care was taken to preserve the developing breast bud architecture.

**Figure 4:**
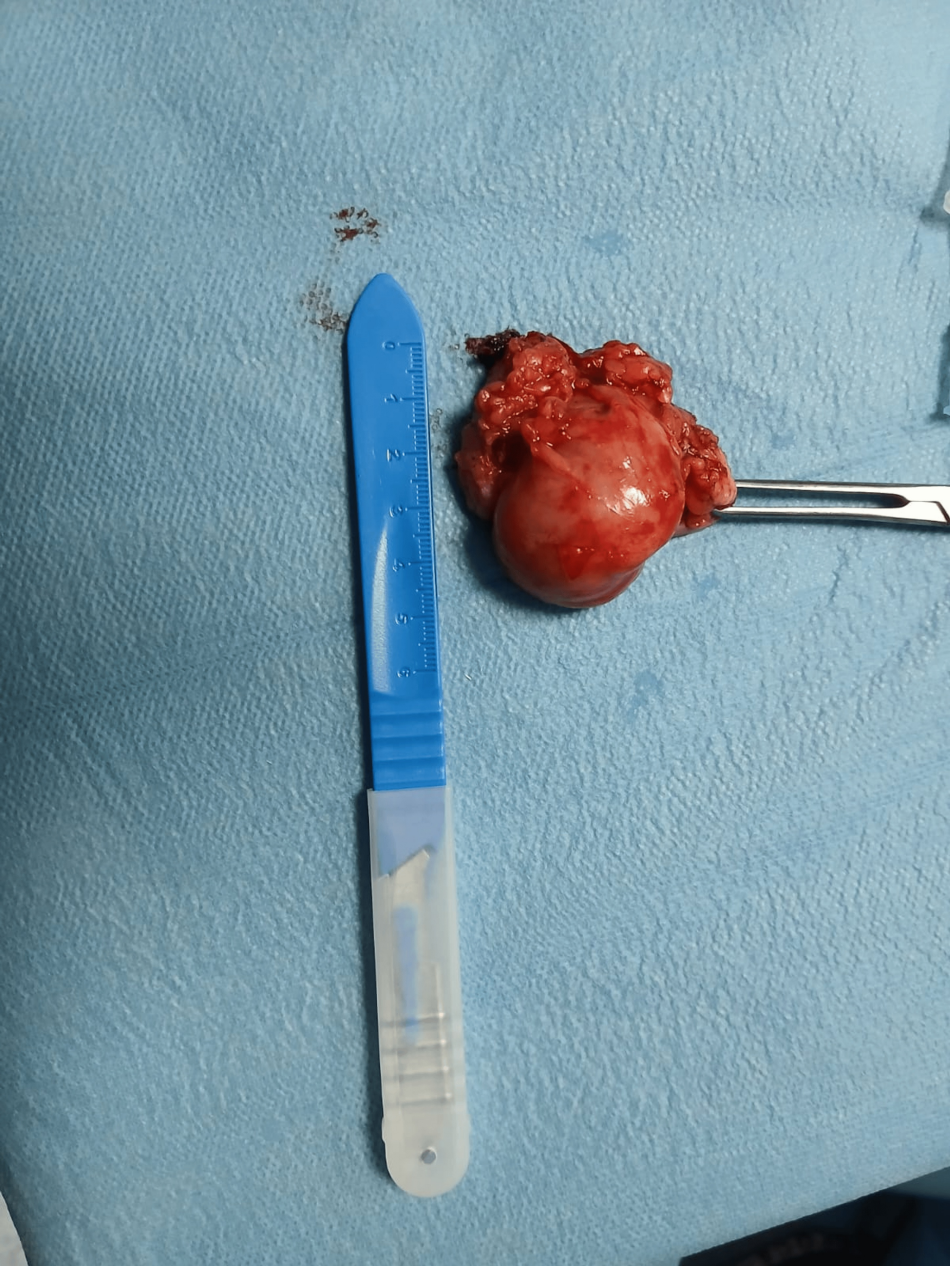
Images showing the specimen sent to anatomopathology

The resected specimen measured 4.5 × 3.2 cm, exhibited characteristic whorled morphology, and was submitted for histopathological analysis (Figure 4). The margins were regular and clearly delineated.

Histopathological Analysis

**Figure 5:**
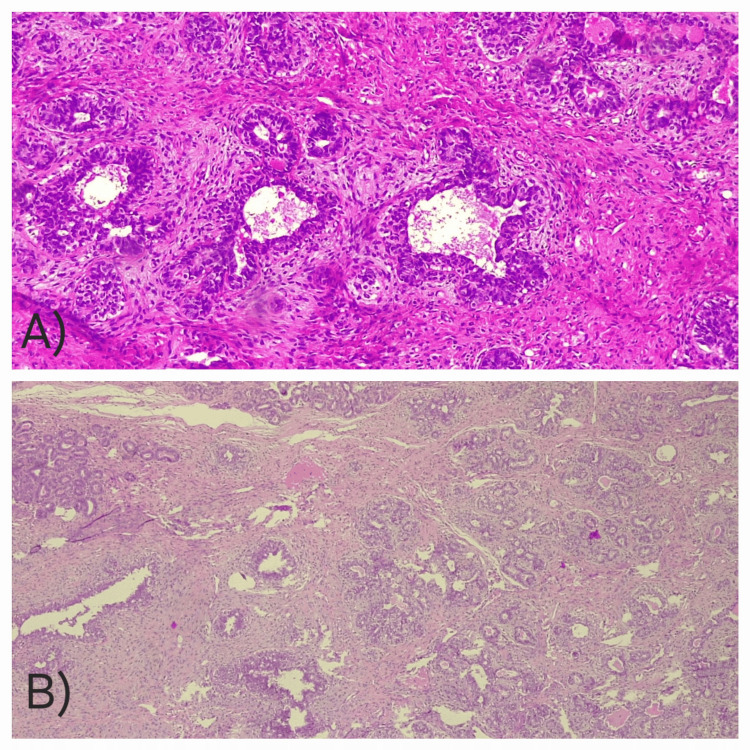
Histopathological features of juvenile fibroadenoma (A) High-power view showing well-formed galactophoric ducts surrounded by fusocellular stroma without atypia or mitotic activity. (B) Low-power view illustrating the overall lobulated architecture with preserved epithelial-stromal organization typical of juvenile fibroadenoma.

The specimen, processed at an accredited private laboratory, demonstrated characteristic features of JF on microscopic examination (Figure 5). The epithelial component showed well-formed galactophoric ducts, while the stromal component revealed dense, homogeneous fusocellular proliferation without atypia or mitotic activity.

Clear surgical margins confirmed complete excision of this benign lesion. Immunohistochemistry (IHC) was not performed and represents a limitation.

Postoperative Course

The patient showed excellent recovery at one-, three-, and six-month follow-ups. There was no evidence of recurrence or surgical complications. Breast symmetry was restored, and both the patient and her family expressed high satisfaction with the outcome.

## Discussion

JF is a rare benign breast tumor that accounts for less than 1% of all fibroadenomas [3]. It is characterized by its rapid growth and large size, often causing significant breast asymmetry and psychological distress in young patients [4]. It is exceptionally rare in prepubertal girls due to underdeveloped breast tissue and limited endogenous estrogen stimulation. Unlike typical fibroadenomas, JF tends to occur in younger age groups, with a peak incidence in adolescents aged 10 to 18 years [5]. The occurrence of JF in a six-year-old girl, as in our case, is exceptionally rare and underscores the need for awareness of this condition in prepubertal children.

This case presents a double paradox: the presence of a hormone-sensitive tumor in a patient with normal estradiol levels and biochemical signs of precocious puberty in the absence of central nervous system pathology or significant estrogen elevation. While Sklair-Levy et al. [6] associate JF with estrogen sensitivity, our findings suggest alternative pathways in prepubertal cases. The preserved tumor encapsulation and cleavage planes, unlike those seen in phyllodes tumors, further support JF’s unique biology in hormonal mosaicism. Clinically, JF presents as a firm, mobile, and painless breast mass, often with rapid enlargement over weeks to months. Imaging studies, including ultrasound and mammography, are essential for initial evaluation. On ultrasound, JF typically appears as a well-circumscribed, hypoechoic mass with homogeneous internal echoes [7]. However, definitive diagnosis requires histopathological examination, which reveals proliferative stromal and epithelial components without atypia [8].

The management of JF depends on the size of the lesion, the degree of breast asymmetry, and the patient's age. Small, asymptomatic lesions may be managed conservatively with close monitoring, while larger or rapidly growing masses often require surgical excision [9]. In pediatric patients, surgical intervention should aim to preserve breast tissue and future breast development, emphasizing the importance of a conservative approach [10].

## Conclusions

JF represents a clinically significant diagnosis in pediatric breast masses, including prepubertal cases. Early detection and proper management are essential for preventing complications and achieving favorable outcomes. JF should be considered even in prepubertal girls with normal estradiol levels. This case emphasizes the importance of considering JF when evaluating breast masses in young girls and demonstrates the value of collaborative care among specialists. Additional research is necessary to elucidate the underlying mechanisms and progression of JF in this unique patient population. Pediatric breast masses, even in very young girls, may represent hormone-sensitive tumors despite normal endocrine profiles. Vigilance and interdisciplinary evaluation are key.
